# The Role of Cell Adhesion Molecule Genes Regulating Neuroplasticity in Addiction

**DOI:** 10.1155/2018/9803764

**Published:** 2018-02-20

**Authors:** Dawn E. Muskiewicz, George R. Uhl, F. Scott Hall

**Affiliations:** ^1^Department of Pharmacology and Experimental Therapeutics, University of Toledo College of Pharmacy and Pharmaceutical Sciences, University of Toledo, Toledo, OH, USA; ^2^Molecular Neurobiology Branch, National Institute on Drug Abuse, Intramural Research Program, Baltimore, MD, USA; ^3^New Mexico VA Healthcare System, Albuquerque, NM, USA

## Abstract

A variety of genetic approaches, including twin studies, linkage studies, and candidate gene studies, has established a firm genetic basis for addiction. However, there has been difficulty identifying the precise genes that underlie addiction liability using these approaches. This situation became especially clear in genome-wide association studies (GWAS) of addiction. Moreover, the results of GWAS brought into clarity many of the shortcomings of those early genetic approaches. GWAS studies stripped away those preconceived notions, examining genes that would not previously have been considered in the study of addiction, consequently creating a shift in our understanding. Most importantly, those studies implicated a class of genes that had not previously been considered in the study of addiction genetics: cell adhesion molecules (CAMs). Considering the well-documented evidence supporting a role for various CAMs in synaptic plasticity, axonal growth, and regeneration, it is not surprising that allelic variation in CAM genes might also play a role in addiction liability. This review focuses on the role of various cell adhesion molecules in neuroplasticity that might contribute to addictive processes and emphasizes the importance of ongoing research on CAM genes that have been implicated in addiction by GWAS.

## 1. Introduction

Substance use disorder (SUD) [1] is a chronic disease characterized by compulsive drug seeking behavior, loss of control of drug intake, and the emergence of negative behaviors resulting from drug tolerance and withdrawal (e.g., anxiety, dysphoria, and other emotional, cognitive, and somatic symptoms [2]). Importantly, this description of SUD includes the persistence of symptoms beyond detoxification. The development of an SUD (herein, we will generally refer to the condition as drug dependence or drug addiction) involves a complex interplay between environmental and genetic factors. However, the genetic component of drug dependence liability is highly polygenic and heterogeneous, with each genetic locus contributing a rather small proportion of the overall genetic variance [3].

Early attempts to characterize the mechanisms underlying addiction liability focused primarily on twin studies, linkage studies, and candidate gene studies. These early studies established that a substantial genetic component contributed to addiction liability through the use of family, adoption, and twin studies, with estimates of the heritability of addiction ranging from 0.3 to 0.7 [4]. However, despite the apparent strength of the heritable component of addiction liability, the early phase of genetic research into the causes of addiction was plagued by a failure to produce a high degree of replication for specific genes or specific gene loci in candidate gene linkage and association studies. There are many reasons for this initial failure, including the often low number of subjects used in many studies, particularly early studies, which is especially problematic if there is a substantial heterogeneity of the underlying genetic architecture, as has been discussed recently [5]. Locus and allelic heterogeneities are less of a problem for GWAS, but of course low numbers of subjects are certainly a problem, as well as low marker density, which also characterized early studies. Over time, the density of markers and the number of subjects included in addiction genetic studies increased, but another important strategy of these postgenomic studies was to look for replication across studies [6–8], the expectation being that if the contribution of each locus was small and heterogeneous it should not be expected that every study would produce identical results, but that positive identification of genes or loci would still reoccur at higher than chance rates across multiple studies in different samples. This did in fact occur. Moreover, analysis of the genes that were repeatedly identified in GWAS produced patterns that were initially unexpected, including a high proportion of CAMs compared to their representation in the genome overall [9, 10] (see Table 1 for the list of CAMs discussed in this review).

After the identification of so many CAMs in GWAS for addiction liability, one of the strategies used to confirm the potential role of these genes as addiction, that is, genes in which variation would affect addiction phenotypes, was to study them in genetically modified mice. Although the human variants likely contributed in more subtle ways to addiction liability, the use of gene knockout (KO) mice in which gene function was eliminated was considered, to some extent, to be a test of whether the identification of particular genes in GWAS was a false positive. Homozygous KO mice are a poor example of human genetic variation, but may still provide information about the potential involvement of genes in addiction. Indeed, given the high degree of genetic heterogeneity that was identified in GWAS, the finding of many positive effects in mice in which these CAMs have been deleted [11] provides strong support for the original GWAS findings as well as the overall concept based on those findings that allelic variation in CAM genes is an important part of the genetic component of addiction liability. Moreover, this suggests that neural plasticity, either during development, or later in life, plays an important role in the genetic component of addiction liability. In considering this idea in this review, we will focus on the potential roles of CAMs in brain function that may be relevant to addiction and then consider the evidence for particular CAMs in addiction.

## 2. Synaptic CAMs

### 2.1. Ig Superfamily

#### 2.1.1. Structure and Function

The immunoglobulin superfamily (IgSF) is the largest and most well documented cell adhesion molecule subgroup, although not all genes in this family are cell adhesion molecules. IgSF contains over 700 genes of which at least 65 proteins are implicated in cell adhesion (for a recent review summarizing cell adhesion molecule classifications, structure, and known functions, see [12], as well as a reassessment of their classifications and potential signaling properties in the nervous system [9]). The Ig superfamily is largely characterized by a variable number of Ig modules, a subregion of the polypeptide chain essential to heterophilic and homophilic binding. Many of the members of the IgSF are built of homologous domains, ranging from 70–110 amino acid residues, with a structure formed by two *β*-sheets packed face-to-face [13]. However, individual members can differ by the number and size of the strands of the two *β*-sheets as well as the conformation of links between them [14]. Ig domains characteristically contain two cysteine residues, placed approximately 55–75 residues apart, and highly conserved tryptophan residues, approximately 10–15 residues downstream of the first cysteine [13]. The extracellular makeup of Ig CAMs can consist exclusively of either several Ig domains connected like beads on a string, as is the case for PECAM-1 and VCAM-1, or Ig connections followed by multiple copies of another molecular building block, such as fibronectin type III (Fn3), as is the case for NCAM and L1 [15]. The FN3 domain is found in most, but not all, IgSF members, and the number of domains often varies between members [13]. Extracellular modules are significantly variable from 1 in P0 to 17 in sialoadhesin. IgSF CAMs can be further subdivided by their membrane anchorage via a glycosylphosphatidylinositol- (GPI-) linked subgroup (e.g., F11, TAG-1, and BIG-1) as well as by their transmembrane subgroup (e.g., neurofascin, NgCAM, L1, and NCAM). Similar to the extracellular components, the cytoplasmic composition of IgSF CAMs also has significant heterogeneity, varying anywhere from 15 to 557 amino acid residues [15].

The wide variation in molecular composition of CAMs on the same general framework suggests their involvement in wide-ranging cellular functions, including different interactions with extracellular and intracellular biomolecules. Alongside indications of involvement of some CAMs in neuroplasticity that will be discussed in this review, members of the IgSF superfamily include many genes involved in immune function and other signaling pathways, including major histocompatibility complex class I and II immunoglobulins, T receptor complex proteins, lymphocyte surface glycoproteins, virus receptors, tumor markers, and growth factor receptors. IgSF CAMs are involved in complex extracellular interactions involving both homophilic and heterophilic binding to CAMs as well as multiple cis and trans interactions [15]. TAG-1/axonin-1, NgCAM, NrCAM, gicerin, DM-GRASP, and NCAM have all been shown to have both homophilic and heterophilic interactions [16]. Several IgSF CAMs have been shown to be involved in axonal growth and guidance during the early development of the nervous system. This involvement is mediated by restricted expression patterns and ability to modulate cell interactions during development, particularly for certain isoforms, and does not exclude roles for the same genes later in life [13].

Our understanding of the physiological roles of CAMs and their interactions with each other and with other intracellular and extracellular proteins, as well as other types of signaling molecules, is still evolving. Indeed, one recent proposal [9], following on a series of clinical and preclinical studies of the role of CAMs in addiction, has completely reassessed the genes that should be classified as CAMs. This study has suggested that there should be a differentiation between CAMs that primarily play a role in information transfer between cells, or between cellular elements and extracellular matrix (“iCAMs”), and those that play primarily structural roles. Furthermore, they subdivided the types of structural CAM classes based on function and location as follows: interactions with cell matrix (mCAMs), tight junctions (tjCAMs), cell-cell interactions in the immune system (cCAMs), focal adhesions (faCAMS), axonal guidance (agCAMs), adherens junctions (ajCAMs), and myelin interactions (myCAMS). It was difficult to make clear distinctions between CAMs that had solely informational and primarily structural roles, and by far, the largest class of CAMs was iCAMs in their analysis. Moreover, this study reassessed gene classifications finding 474 likely CAM genes, of which 283 would be classified as iCAMs. Many of those are discussed in more detail below. This analysis supports previous emphases on the signaling aspects of synaptic formation played by several classes of CAMs [17].

#### 2.1.2. NCAM1

**Figure 1:**
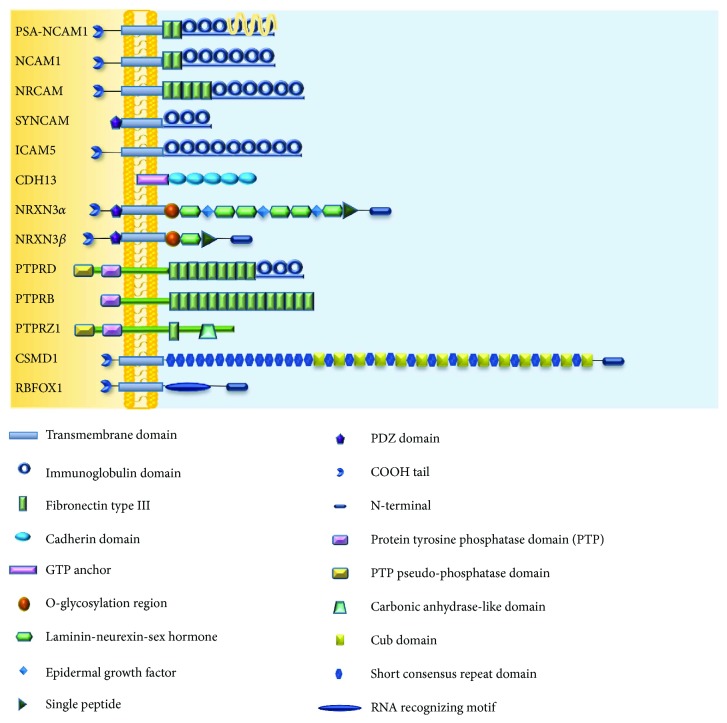
Schematic representation of the structural motifs of cell adhesion molecules discussed in this review.

Neural cell adhesion molecule 1 (NCAM1; see Figure 1 for a comparison of the structure of this CAM to other CAMs discussed in subsequent sections) is expressed across many cell types, including neurons, glial cells, cardiac muscle cells, and skeletal muscle cells, with as many as 27 distinct isoforms generated by alternative RNA splicing [15]. In general, NCAM can be expressed both pre- and postsynaptically and has three distinct classes of isoforms including transmembrane linked (seen in Figure 1), GPI anchored, and secreted or soluble NCAM. NCAM is composed of five Ig domains, encoded by two exons, and two fibronectin type III domains [18]. The role of NCAM1 in the brain was first characterized by Hoffman et al. [19], where it was shown to mediate retinal cell adhesion. Since then, NCAM1 has been shown to have roles in axonal development, involvement in signaling pathways, emotional function, and learning, as well as potential involvement in many neuropsychiatric and neurodegenerative disorders [20–25, 15]. More recent studies have indicated a role of NCAM1 in addiction, specifically the polysialylated form of NCAM1 (PSA-NCAM1), which commonly regulates the adhesive properties of the molecule and is critical for effects of NCAM on synaptic plasticity [25, 26]. The addition of the long linear homopolymers of alpha-2,8-linked sialic acid residues to NCAM1 produces antiadhesion properties. NCAM has been shown to mediate both pre- and postsynaptic scaffoldings that influences excitatory synapse formation and plasticity relevant to addiction. Studies have revealed that NCAM can effect postsynaptic scaffolding associated with *β*-spectrin and accumulation of PSD95, GluN1, GluN2B, and CaMKII [27]. In addition, PSA-NCAM was shown to increase AMPAR-mediated currents, although this was age dependent [28]. PSA-NCAM was also shown to affect NMDA receptor activity by inhibiting receptor currents in cultured hippocampal neurons at low, but not high, concentrations of glutamate, suggesting a role as a potential competitive antagonist at the glutamate binding site [29]. Given its substantial interaction with glutamate excitatory synapses, it is not surprising that PSA-NCAM has also been found to play a substantial role in many behavioral tests of addiction that involve the formation of drug-dependent memories. For instance, single cocaine administration decreases the number of PSA-NCAM1-positive neurons in the dentate gyrus (DG) of male Wistar rats, as well as decreasing the length of PSA-NCAM1-positive dendrites [30]. Similarly, amphetamine was shown to decrease the expression of 180–200 kDa isoform of PSA-CAM in the hippocampus of male C57BL/6 mice, although this appeared to occur regardless of whether drug exposure was specifically paired with a distinctive environment or not [31]. Thus, the role of PSA-NCAM1 may be specific to certain experimental circumstances not represented by the locomotor sensitization approach. Additional evidence supports a role of PSA-NCAM1 in other learning contexts. The cannabinoid receptor 1 agonist HU-210, an illicitly used synthetic cannabinoid, also antagonizes hippocampal synaptic plasticity associated with contextual fear conditioning, an effect that involves reversing the increases in PSA-NCAM1 expression involved in that type of learning [32]. Nicotine self-administration also reduces levels of PSA-NCAM1 in the DG of rats, in a dose-dependent manner [33]. PSA-NCAM1 levels in the ventromedial prefrontal cortex (vmPFC) are associated with the ability to transfer learning of a classically conditioned association to instrumental learning (Pavlovian-to-instrumental transfer (PIT)), and reduction in vmPFC PSA-NCAM1 levels by administration of endoneuraminidase reduced extinction behavior in a PIT task for ethanol reinforcement [34]. This study not only demonstrates the importance of PSA-NCAM1 in ethanol reinforcement, but also demonstrates its involvement in a specific behavior that has high relevance to drug dependence—extinction of drug reinforcer-mediated behavior.

In contrast to many of the findings discussed above, a clinical study found that PSA-NCAM1 levels were increased in the DG of heroin-addicted individuals [35]. The effects of opiates on *NCAM1* expression have not been studied, but presuming the acute effects of opiates are similar to those of other addictive drugs, this increase in PSA-NCAM1 might reflect a compensatory process associated with the repeated downregulation produced by acute effects of the drug, or an effect associated with drug withdrawal. As an additional piece in this still incomplete puzzle, neonatal nicotine exposure was found to reduce levels of *Ncam1* mRNA in the amygdala of female rats when assessed in early adolescence [36]. This study will be discussed in more detail throughout this review as it specifically addressed the effects of prenatal nicotine on the expression of a number of CAMs.

It is obvious that further studies are needed to fully elucidate the potential roles of NCAM1 in addictive processes under different conditions, including in response to different types of addictive drugs, at different ages and at different parts of the addiction cycle. However, it should be noted that the majority of the findings discussed in this section do not reflect changes in the expression of NCAM itself, but rather the levels of PSA-NCAM. Moreover, genetic associations for *NCAM* with drug dependence were not found in the majority of GWAS studies previously discussed, although *NCAM* has been recently associated with marijuana dependence [37]. One study did find that genes near *NCAM1* were associated with nicotine dependence [38]. However, this is a complex genomic area where *NCAM1* is part of a gene cluster with *TTC12*, *ANKK1*, and *DRD2*, and stronger associations have been found for markers within the other genes in this cluster [39, 40]. Nonetheless, an analysis of this region using a family-based association approach with denser marker coverage found an association of markers near the *NCAM1* exon 12/intron 13 border with alcohol dependence [41]. Although NCAM1 (as PSA-NCAM1) may have a role in certain aspects of the addictive process there is, thus far, less evidence that allelic variation in the *NCAM1* gene contributes to addiction liability. This may be because *NCAM1* allelic variation contributes more to certain aspects of the addictive process, certain addiction phenotypes, or to addiction to particular substances. Indeed, a previous review of this subject has suggested that, although GWAS for drug dependence have been successful, this may be too broad of a phenotype and that stronger effects will be found for more specific drug addiction phenotypes [5]. It is also highly possible that only particular splice variants are associated with addiction, rather than *NCAM1* overall; a topic that will be considered again with respect to *A2BP1* (ataxin-2 binding protein 1).

#### 2.1.3. NRCAM

*NRCAM* (neuronal cell adhesion molecule or *NgCAM*-related cell adhesion molecule; see Figure 1) belongs to the L1 family of IgSF CAMs and is composed of six Ig-like domains, five FN3 domains in its extracellular region, and a cytoplasmic region composed of approximately 110 amino acid residues [42]. NRCAM can interact with molecules both intracellularly and extracellularly. Several studies have indicated that the extracellular domain of *NRCAM* can interact with molecules on the cis- and transmembranes, as well as both homophilic and heterophilic binding with CAM and CAM-like molecules. *NRCAM* has been implicated in axonal growth and guidance, playing an important role in the development of cerebellar granule cells, dorsal and ventral spinal cord axonal development, optic chiasm formation, and the formation of thalamocortical projections [43].

Given its role in the development of thalamocortical projections and its general distribution in areas thought to be important in addiction [44], it is not surprising that *NRCAM* was hypothesized to play a role in addiction. Relationships between markers in or near the *NRCAM* gene and drug or alcohol dependence were found in genome-wide linkage studies for alcoholism [45–47] and early genome-wide association studies for drug dependence [48, 49]. Based on these findings, candidate gene approaches were used which found that *NRCAM* allelic variants were associated with drug dependence [44] and methamphetamine dependence [50]. In later GWAS studies, utilizing more subjects and greater marker density, *NRCAM* allelic markers were also associated with caffeine intake (Amin et al., 2012). However, the majority of later GWAS studies did not find significant associations of *NRCAM* variants with drug dependence. This may be because *NRCAM* variants are more closely related to particular endophenotypes that were better represented in some samples; in addition to an association with methamphetamine dependence, Yoo et al. [50] also found that *NRCAM* allelic markers were associated with specific measures of addictive behavior and personality traits thought to be a characteristic of drug abusers, including novelty seeking.

As discussed above, one of the reverse translational approaches used to confirm the possibility that variation in the *NRCAM* gene may contribute to addiction was the use of *Nrcam* KO mice. The logic behind this approach was specific; Ishiguro et al. [44] demonstrated that the same *NRCAM* markers associated with drug dependence were also associated with a 74% reduction in *NRCAM* expression. There was also a substantial upregulation of *Nrcam* gene expression after morphine treatment in rats. Thus, it might be expected that individuals with poorer expression might respond quite differently when taking drugs of abuse. On this basis, *Nrcam* KO mice were examined for condition place preference ((CPP) a measure of the reinforcing efficacy of drugs) produced by several drugs of abuse, including morphine, cocaine, and amphetamine. Reduced CPP was observed in both heterozygous and homozygous *Nrcam* KO mice. In a two-bottle free-access ethanol consumption paradigm, it was also found that male *Nrcam*+/− mice display reductions in ethanol consumption compared to wild-type littermates [51]. Furthermore, studies have shown that *Nrcam* KO mice have no general learning impairments that might confound CPP studies [44, 52]. However, other behavioral differences were observed, including a passive avoidance deficit interpreted as a result from poor impulse control [52]. Although the interpretation of that test is not certain, behavior seen in tests of responses to anxiety and novelty [51] could be interpreted in a similar manner. Certainly, examination of these mice in a circumstance that more specifically assesses impulsive behavior is warranted, although the general findings are consistent with those of Yoo et al. [50] in methamphetamine addicts.

#### 2.1.4. Synaptic Cell Adhesion Molecules (SYNCAMs)

The idea, developed from the results of a series of GWAS studies discussed above, that differences in the function of certain CAMs may contribute to addictive behavior has opened the door for the study of several other CAMs that were not implicated in GWAS studies. Obviously, from the name, the synaptic cell adhesion molecules ((SYNCAMs); see Figure 1) have prominent roles in synaptic cell adhesion, and synapse formation [53], as well as axon guidance during development [54]. SYNCAMs are IgSF/SYNCAM proteins that span the synaptic cleft and induce the formation of excitatory synapses *in vitro* [55], a process seen previously only with neuroligin [56]. Like NCAM1, polysialylation of SYNCAM1 is important to its function, with polysialylation of SYNCAM in the first Ig domain preventing homophilic binding [57]. As the formation of excitatory synapses has long been thought to be an important part of addictive processes, demonstrated by dendritic spine formation in several brain regions after exposure to drugs of abuse [58–60], along with increased surface expression of AMPA receptors [61–63], it would be natural to investigate SYNCAM1. Moreover, SynCAM1 was already known to influence synaptogenesis in the hippocampus and to be ubiquitously expressed throughout much of the brain, including the striatum and other structures know to exhibit synaptic plasticity after exposure to drugs of abuse. Consistent with this hypothesis, *Syncam1* KO mice showed decreases in the length of mushroom dendritic spines as well as impaired locomotor sensitization to cocaine [64].

However, with regard to the genetic basis of drug dependence liability, there is little evidence for contributions from allelic variation in *SYNCAM1*, judging from the GWAS studies mentioned above. A related family member, *CADM2*, was associated with marijuana dependence [37], but has not otherwise been investigated regarding the effects drugs of abuse or addiction.

#### 2.1.5. Intercellular Adhesion Molecule 5 (ICAM5)

ICAM5 (see Figure 1) is an immunoglobulin cell adhesion molecule highly expressed on the dendrites of neurons in the telencephalon that is known to have roles in immune and neural functions [65, 66]. Moreover, *in vitro* studies have indicated that the soluble form of ICAM5 may regulate glutamatergic neurotransmission [67, 68]. Not much is known about the ability of ICAM5 to affect addictive behavior through influences on glutamate function, but one study has shown that acute methamphetamine (MA) treatment stimulates cleavage of membrane-bound ICAM5 molecules, both *in vitro* and *in vivo*, via matrix metalloproteinase 9 (MMP9) [69]. *Mmp9* has been shown to have roles in alcohol seeking behavior and methamphetamine toxicity in rodents [70, 71]. Moreover, matrix metalloproteinases (and MMP9 in particular) have been suggested to affect neural plasticity and learning relevant to drug addiction [72, 73].

Genetic variation in ICAM5 has not been found to be associated with drug dependence in GWAS studies, and has not been a focus of other genetic approaches, although MMP9 has been associated with alcohol dependence [74], and was identified in the brains of cocaine abusers in a transcriptional study [75]. Based upon the role of ICAM5 in altering glutamatergic neurotransmission, including changes in response to methamphetamine, further research is warranted. The case of ICAM5, has an important implication: although some proteins may have roles in the circuitry underlying the effects of drugs of abuse, there may, for whatever reason, not exist genetic variation in the human genome that contributes to the genetic liability toward drug dependence.

### 2.2. Cadherins

#### 2.2.1. Structure and Function

The cadherin superfamily of CAMs, consisting of more than 110 members, are transmembrane proteins defined by a repeated extracellular domain sequence, the cadherin EC domain [76, 77]. Each EC domain consists of seven *β*-strands, each forming two *β*-sheets, folding similarly to that of immunoglobulin domains, that play important roles in brain morphogenesis and wiring [76]. The stabilization of the extracellular domain in cadherins relies upon the presence of Ca^2+^, which binds to the boundaries between EC domains, resulting in the formation of its rod-like structure [78]. EC domains containing several conserved Ca^2+^ binding sequences in cadherins include AXDXD, LDRE, and DXNDN domains. Cytoplasmic interactions of cadherins are essential to their cell-cell adhesion process [79]. Catenins have interactions with the cytoplasmic domain of cadherin molecules, and thus interactions between catenins and cadherins are important to their function [80–82]. These molecules included three different groups, *α*, *β*, and p120, which are essential in mediating linkage of the cytoplasmic components of cadherin molecules to the actin cytoskeleton of the cell. “Classical cadherins” have five EC domains, but the cadherin superfamily includes proteins with other numbers of domains as well (see [76] for a complete review of cadherin subtypes and classifications).

A number of classification schemes for cadherin have been proposed, but one clear division is between classical and nonclassical cadherins [76]. Nonclassical cadherins include a large number of cadherins, including CDH13, desmosomal cadherin, 7D family protocadherins, CDH23, fat-dachsous, CDH26, CDH28, flamingo, calsyntenins, and RET, which differ in a number of structural aspects, while still conforming to the basic cadherin structure. The size of cadherins differs substantially among nonclassical cadherins, particularly in terms of the size of the extracellular and intracellular domains. Structural variations in cadherins include differences in the number of cadherin (EC) motifs, as well as the presence/absence or number of several other intracellular and extracellular motifs, including the *β*-catenin binding site, p120 binding site, desmoglein repeats, and intracellular kinase domains. Classical cadherins belong to a group of type I transmembrane proteins containing five EC domains and a unique cytoplasmic domain. In humans, this class of cadherins consists of 18 members that are highly conserved and interact with *β*-catenin and p120-catenin. Classical cadherins can be further subdivided into the type I classical cadherins consisting of CDH1 (E-cad), CDH2 (N-cad), CDH3 (P-cad), CDH4 (R-cad), and CDH15 (M-cad); the type II classical cadherins include CDH5 (VE-cad), CDH6 (K-cad), CDH7, CDH8, CDH9 (T1-cad), CDH10 (T2-cad), CDH11 (OB-cad), CDH12 (N-cad-2), CDH18, CDH19, CDH20, CDH22, and CDH24.

Cadherins are thus a diverse class of molecules involved in many physiological functions, with diverse tissue-specific distributions [76]. Cell-cell interactions and cell adhesion are essential for the development of multicellular organisms, and especially in the development and plasticity of many complex organs such as the central nervous system. In particular, the formation of neural networks involves a series of processes that include cell fate determination, proliferation, migration, differentiation, axon elongation, pathfinding, target recognition, and synaptic plasticity, many of which rely on cell-cell adhesion and interaction. One of the more overlooked aspects of neural network formation and plasticity, however, is that many of these processes also involve cell signaling, which has been proposed to be an important criterion of CAM classification [9]. Cadherins have been implicated in a wide variety of these cellular processes that contribute to development and plasticity (for review see [76]). Certainly, not all cadherins may be involved in processes relevant to addiction, which means that those that do specifically play roles in addiction may constitute targets for antiaddiction drug development [10]. As an example of the specificity of the roles of these cadherins in neural function, the protocadherin class appears to regulate aspects of neural cell identity and diversity [83], rather than neuroplasticity. In the following section, only CDH13 will be considered, as it is the only cadherin for which there is strong evidence of a role in addiction, based upon neurobiological and genetic studies.

#### 2.2.2. Cadherin 13 (CDH13)

*CDH13* (see Figure 1) is one of the genes that has been most often found to be associated with drug dependence or other addiction phenotypes in GWAS [6, 84–95]. Although many of these findings involve dependence on particular addictive substances, or nicotine cessation, others involve general drug dependency, or responses that may be involved in the broad category of drug dependence. Prior to observation of these relationships in GWAS, there was no interest in CDH13 in the addiction field. However, CDH13 is glycosylphosphatidylinositol-anchored cell adhesion molecule, prominently expressed by ventral tegmental area and substantia nigra pars compacta dopamine neurons [96, 97], which are commonly associated with reward, locomotor control, and cognitive functions.

Some of the genetic markers used in association studies described above were also found to be associated with levels of CDH13 gene expression in human postmortem brain samples [98]. Using the same logic as was previously described for NRCAM [44], *Cdh13* KO mice were used to examine the effects of alterations in *Cdh13* expression on addiction-related phenotypes [98]. Firstly, cocaine CPP was examined in constitutive *Cdh13* KO mice, which showed evidence for a leftward shift in the dose-response curve—increased preference at 5 mg/kg s.c. and reduced preference at 10 mg/kg s.c. The reduction in cocaine CPP at 10 mg/kg was observed in both *Cdh13*+/− and *Cdh13*−/− mice. Furthermore, the same increase in preference for a low dose of cocaine was observed in conditional *Cdh13* KO mice, in which the transgene was activated in adulthood, thus negating the possibility that the effects in the constitutive KO mice were due to developmental effects.

For all of the CAMs discussed here, one of the most fundamental questions is whether the role that genetic variation plays in addiction liability is due to altered synaptic plasticity during development or in adulthood. The conditional knockout study strongly suggests that it is adult neuroplasticity that is affected. This argument was further assessed in (previously unpublished) gene expression data discussed below.

### 2.3. Neurexins/Neuroligins

#### 2.3.1. Structure and Function

Other groups of CAMs that have been implicated in GWAS for addiction are neurexins (NRXNs) and neuroligins (NLGNs) [99], two classes of membrane-bound proteins involved in the central organization of glutamatergic and GABAergic synapses [100, 101], that have been implicated in a variety of neurodevelopmental disorders. This class of genes has been well described for some time, and their role in synapse formation has been well elaborated (for a more complete description see [102]). Briefly, in mammals, there are three neurexin genes, each with five alternative splicing sites, and three known extracellular binding partners (neuroligins, dystroglycan, and neurexophilins). Each gene has an upstream promoter, generating *α*-neurexin, and a downstream promoter which generates a smaller *β*-neurexin. Neurexins contain laminin, neurexin, and sex hormone-binding protein (LNS) domains which differ in number between *α* and *β* variants in addition to a highly glycosylated region, a transmembrane domain, and PDZ binding domain (PDZ-BD). Similar to neurexins, neuroligins are composed of a highly glycosylated region, a transmembrane region, and a PDZ-BD; however, their main extracellular domain is composed of a region homologous to acetylcholinesterase, but lacking cholinesterase activity. Neurexins are thought to localize to the presynaptic terminus and trigger postsynaptic differentiation while their binding partner, neuroligin, is thought to perform the opposite function, contributing to presynaptic differentiation via postsynaptic localization [103]. These CAMs are therefore thought to play an important role in synaptogenesis, and studies have shown that overexpression of neuroligins increases the number of synapses formed [104]. This role in synaptogenesis is not thought to be an exclusive role of these molecules, but to involve a number of CAMs [17]. Moreover, it would appear that specific CAM isoforms are involved in forming synapses in particular neural circuits (as well as initial circuit formation). This possibility of more specific roles in distinct brain regions has important implications for the potential roles of CAMs in addiction (as well as other functions and dysfunctional states).

#### 2.3.2. Neurexin 3 (NRXN3) and Neuroligin 1/2/3 (NLG1/2/3)

*NRXN3* (see Figure 1) has recently been implicated in addiction by GWAS studies of drug and nicotine dependence [99, 105], genome-wide linkage for opioid dependence [106], and with candidate gene approaches for alcohol, nicotine, or drug dependence [107–109], including smoking in schizophrenia patients [110]. Moreover, and again using the previous strategy of examining postmortem human tissue expression, in human studies revealing an association with a SNP potentially altering *NRXN3* gene splicing (rs8019381, located 23 bp from splicing site 5) and alcohol dependence, individuals with the addiction-associated rs8019381 T allele showed significantly lower levels of transmembrane NRXN3 isoforms [108]. There is also potential evidence for a relationship of *NRXN3* markers to addiction endophenotypes, including impulsivity [109].

As stated previously, NRXNs and NLGNs have important actions on both pre- and postsynaptic scaffolding and affect synaptic plasticity. Of importance for addiction phenotypes, these CAMs affect synaptic functions on both excitatory glutamate synapses as well as inhibitory GABA synapses. For instance, NRXN1*β* drives functional postsynaptic assembly of NMDARs and AMPARs on hippocampal neurons [111, 112]. Increased NLGN2 expression has also been found to increase GABAergic and glycinergic transmission, while NRXN*β* has been shown to decrease GABA_A_R-mediated transmission through extracellular binding to GABA_A_ *α*R1 [113, 114]. The role of NRXNs and NLGNs in the development of addictive behaviors has also been examined in rodent studies, although to a limited extent. C57BL/6J mice, a strain commonly used in addiction studies due to their high levels of self-administration of most drugs of abuse, have lower levels of *Nrxn2β* and *Nlgn3* expression in the substantia nigra and increased expression of *Nlgn1* in the subthalamic nucleus compared to non-drug-preferring mice [115]. That same study also found that cocaine conditioning in a CPP procedure increased the expression of *Nrxn3β* in the globus pallidus. The combined human and animal data offer compelling evidence to support *Nrxn3* dysregulation as a potential mechanism contributing to addictive disorders. However, further research is needed in both humans and animal models to solidify this potential role of NRXN3, and *NRXN3* genetic variance, in drug dependence.

### 2.4. Other CAM Classes

There are other CAM genes, from other CAM classes besides those discussed above, that have also been associated with addiction [8, 10]. These findings include the genes for several protein tyrosine phosphatase receptor type cell adhesion molecules ((PTPR); see http://www.genenames.org/cgi-bin/genefamilies/set/813 for a discussion of the nomenclature for this complex gene family), including *PTPRD* and *PTPRB*, as well as CUB and Sushi multiple domains 1 (*CSMD1*). Also included in this group of genes is *A2BP1*, which although it is not a cell adhesion molecule itself, has an important role in RNA splicing of cell adhesion molecules, including many discussed here that have been associated with drug dependence. PTPRs have both cell adhesion and catalytic activity that varies substantially across family members [116].

#### 2.4.1. PTPRD

Many of the GWA studies discussed previously in this review identified clusters of SNPs with nominally significant associations (10^−2^ > *p* > 10^−8^) with drug dependence and addiction-related phenotypes [6–8, 85, 88, 91, 92, 117–120]. Although the magnitude of the association in many of these studies did not reach “genome-wide significance,” the repeated identification of an association in multiple samples suggested that this was indeed a real association, but with a small effect size as part of a highly polygenic genetic architecture. Subsequently, another laboratory has also found an association between *PTPRD* markers and opiate dependence in a GWAS for copy number variants in opiate-dependent individuals [121]. In a general way, these findings are consistent with the brain distribution of *PTPRD*, which is prominently expressed in ventral midbrain neurons implicated in reward, locomotor control, and sleep processes [122]. PTPRD forms both homodimers involved in the formation of neurites [123] and heterodimers, including PTPRD/SLITRK3 heterodimers that are involved in GABAergic synaptic plasticity [124]. Interestingly, SLITRK3 is from a family of Slit- and Trk-like proteins classified as “synaptic organizers.”

*PTPRD* addiction-related haplotypes were shown to correlate with mRNA levels in human brain samples [125], providing the same sort of logic for examining drug responses in *Ptprd*-deficient mice (e.g., *Ptprd* KO mice) as for *Nrcam* and *Cdh13*. A leftward-shifted dose-response relationship for cocaine reward was observed in *Ptprd*+/− mice [125]. Heterozygous PTPRD KO displayed greater preference for places paired with 5 mg/kg cocaine as opposed to places with 10 or 20 mg/kg [125]. By contrast, cocaine preferences in *Ptprd*−/− mice were reduced at all doses. Obviously, much remains to be done in order to determine the role of PTPRD in response to drugs of abuse and for *PTPRD* variation in the genetic liability for drug dependence; a subsequent section presents additional data supporting this relationship based on cocaine regulation of *PTPRD* expression.

#### 2.4.2. PTPRB

Protein tyrosine phosphatase receptor-type beta (*PTPRB*) is a part of the larger family of PTPRs which are known to regulate a variety of cellular processes including cell growth, differentiation, mitosis, and oncogenic transformation [126]. PTPRB contains extracellular fibronectin domains that interact with cell adhesion molecules such as contactin. To date, only a single study implicated PTPRB in substance abuse, finding significant associations for two *PTPRB* polymorphisms with alcoholism vulnerability in unrelated European-American individuals [127]. Additionally, mouse studies found that levels of *Ptprb* in the caudate putamen, midbrain, and hippocampus of C57BL/6J mice were significantly increased after both acute and chronic exposure to 20 mg/kg of morphine. It is obvious that further investigation is needed to elucidate and confirm the potential role of PTPRB in the neuroplasticity of addiction.

#### 2.4.3. PTPRZ1

*Ptprz1* (also called RPTP*β*/*ζ*) is upregulated by acute morphine treatment and downregulated after chronic treatment in rodents [128]. Moreover, the PTPRZ1 ligand pleiotrophin [129] was also acutely upregulated by acute morphine treatment, but levels were normalized after chronic treatment, and upregulated by naloxone-precipitated withdrawal. These effects apparently involve signaling between astrocytes, which had elevated pleiotrophin expression, and midbrain dopamine neurons expressing PTPRZ1. Pleiotrophin is also upregulated by cocaine and amphetamine [130, 131] and may be involved in the extinction of cocaine-conditioned responses [132] and opiate withdrawal [133]. Adolescent amphetamine disruption of adult hippocampal plasticity is also dependent on pleiotrophin [134]. Some of these effects may be involved in the neurotoxic effects of these drugs as well [135–137].

Despite the accumulating evidence for a role of *Ptprz1* in addiction-related phenotypes from preclinical models, this gene was not identified in GWAS for addiction-related phenotypes. As mentioned before, there could be numerous reasons for why genetic variation in this gene in humans does not exist or has not been found to contribute to addiction-related phenotypes, not the least of which is the potential complexity of genetic contributions to addiction liability. Another PTPR family member, PTPRG, does not produce significant associations on its own with addiction-related phenotypes, but significant epistatic interactions of PTPRG markers with other genes were found in a recent GWAS examining alcohol dependence symptom counts [138].

#### 2.4.4. CSMD1

CUB and Sushi multiple domains 1 (*CSMD1*) is a multiple domain complement regulatory protein that is highly expressed in the central nervous system [139]. CSMD1 consists of 14 CUB domains separated by short consensus repeat (SCR) domains (also called Sushi repeat domains), followed by 15 tandem SCR domains. Like many other cell adhesion molecules, CSMD1 is a type 1 membrane protein spanning the membrane once. It is enriched in nerve growth cones [139], and its cellular tissue distribution in the adult brain includes the ventral midbrain (Allen Mouse Brain Atlas), making it likely to be involved in processes associated with drug dependence. Indeed, *CSMD1* is among the most highly replicated genes for drug dependence and related addiction phenotypes [6–8, 87, 88, 91, 92, 117–120]. Moreover, in a recent large GWAS study of cannabis dependence, a *CSMD1* marker was found to be associated with cannabis dependence at a genome-wide level of significance [140]. In a study of psoriasis, for which smoking is a risk factor, *CSMD1* variants were found to be associated with smoking and psoriasis in an interactive fashion [141]. In a similar manner, *CSMD1* copy number variants were found to be interactively associated with alcohol consumption as a risk factor for head and neck squamous cell carcinoma [142]. The mechanisms by which *CSMD1* variants may influence drug dependence liability are unknown and likely to be a part of broader effects on brain function as *CSMD1* markers have been associated with schizophrenia [143], autism [144], bipolar disorder [145], and general cognitive ability and executive function [146]. This later finding may suggest that the role of *CSMD1* variants in all of these conditions may result from impairments in executive function and decision making.

As with other genes considered here, one of the approaches taken to consider the role that *CSMD1* may have in drug dependence was to examine the effect of its removal in mice. A *Csmd1* KO strain was created by Lexicon Pharmaceuticals in which the first exon was deleted and has been described in three studies to date. In the first study, no differences in any behavioral phenotypes relevant to schizophrenia were observed, including tests of prepulse inhibition of acoustic startle, social interaction, sucrose preference, and locomotor activity [147]. In a second study, *Csmd1* KO did produce changes in measures of affective behavior indicative of anxious and depressive phenotypes [148]. In the final study, homozygous *Csmd1* KO mice had impaired learning of the Morris water maze [149]. More importantly, for the present discussion, that study also found subtle, but significant, reductions in cocaine CPP in both heterozygous and homozygous *Csmd1* KO mice. In human postmortem brain samples, *CSMD1* variants were associated with differential gene expression, as has been found for several of the genes previously discussed here.

Although certainly encouraging, much more remains to be explored in order to confirm and extend the findings in genetically modified mice of phenotypes consistent with human clinical findings. Moreover, although it is quite logical based on the limited data available to hypothesize that *CSMD1* variation is influencing the development or plasticity of neural circuits relevant to these phenotypes, this remains to be studied in a specific manner.

#### 2.4.5. RBFOX1

RNA binding protein fox-1 homolog (ataxin-2 binding protein 1 (A2BP1)) binds to the C-terminus of ataxin-2 [150, 151], but also has RNA-binding motifs recognizing the RNA element (U)GCAUG and is involved in alternative splicing [152] of many genes expressed in neural cells [153]. Moreover, the target specificity of RBFOX1 is tissue dependent [154]. CNS-specific deletion of *Rbfox1* in mice increases neuronal excitability in the dentate gyrus and produces spontaneous seizures [155]. Few changes in overall transcript expression were observed in these mice, but there were substantial changes in the relative abundance of particular transcripts, including genes involved in membrane excitability (*Gabrg2*, *Grin1*, *Scn8a*, and *Snap25*), but also the CAMs *Nrcam* and *Nrxn3*. The inclusion of these CAMs, in addition to alterations in neuronal excitability, would seem to indicate that RBFOX1 may affect synaptic plasticity as well as neuronal excitability. Indeed, the network of transcripts regulated by RBFOX1 has been implicated in the organization of neural circuits during development [156], particularly in the forebrain [157], and has been implicated in autism spectrum disorder in genomic and transcriptomic studies [158, 159].

Of particular relevance here, *RBFOX1* markers have been repeatedly associated with drug dependence and related phenotypes [6, 85–89, 92, 117, 119, 120, 160, 161], findings also supported by linkage analyses [47, 162, 163]. In support of these genetic findings, cocaine treatment has been found to substantially affect alternative splicing, effects hypothesized to involve RBFOX1 [164].

### 2.5. Regulation of CAM Expression by Cocaine

The specific role of CAMs discussed here in addiction and addiction phenotypes is not fully known. In particular, for the majority of these genes (except perhaps for *CDH13*), it is not known whether the role of polymorphisms is to influence CAM expression during development, or neural plasticity in response to exposure to drugs of abuse. If the primary role of CAMs is in neural plasticity occurring in response to drugs of abuse, it would be expected that drugs of abuse would alter the expression of the CAMs that GWAS (and mouse genetic studies) have shown are important for addiction liability and addiction phenotypes. Here, we report preliminary evidence that many of these CAMs are regulated by cocaine. The effect of cocaine on the expression of *CDH13*, *CSMD1*, *PTPRD*, and *A2BP1* transcripts was examined using rtPCR (see supplement for detailed methods). Multiple transcripts were examined (see Supplemental Table 1 for descriptions). Gene expression was examined in tissue samples (striatum, hippocampus, frontal cortex, and ventral midbrain) under two conditions: after a regimen of repeated cocaine injections used to induce locomotor sensitization and after the cocaine treatment regimen used to induce cocaine-conditioned place preference. Both treatment conditions produced changes in CAM expression, although the pattern was somewhat different. Because this study is highly preliminary, the data is presented with uncorrected *p* values. Some of these are likely to represent false positives (those with *p* values between *p* < 0.005 and *p* < 0.05); something that will need to be addressed in additional, more comprehensive, studies.

Despite this, the tentative nature of these findings, the whole data set, representing examination of 4 genes chosen from the entire genome based on the GWAS and mouse genetic studies described in this review, provides strong evidence for the importance of alterations in the expression of these genes in response to cocaine. Although dependent on brain region, alterations in at least one transcript were found for all 4 genes after exposure to sensitizing regimen of cocaine (Table 2). In the striatum, cocaine increased the expression of *CDH13a*, *CSMD1g*, and *PTPRDd*. Increases in the expression of *PTPRDd* were also observed in the ventral midbrain and *CDH13a* in the cerebral cortex. Reductions in the expression of *RBFOX1a* and *PTPRDa* were observed in cerebral cortex. No changes were observed in the hippocampus. These changes were not large in magnitude, but as they would be expected to occur only within particular cell types, influencing particular synaptic connections, this is not surprising.

Even more changes were observed after conditioned place preference (Table 3). The expression of *RBFOX1a* was decreased in the striatum, as were the levels of *PTPRDd*. In the cerebral cortex, the levels of *CDH13a* were again increased, as they were after noncontextual cocaine treatments. The levels of *CSMD1g* and *CSMD1h* were also increased in cerebral cortex. *CDH13a* levels were also increased in the ventral midbrain, as were levels of *CSMD1g*. Perhaps, consistent with the greater contextual and spatial learning associated with the CPP procedure, changes in the expression of several CAMs were observed in the hippocampus, in contrast to what was observed after a simpler sensitization procedure. Reduced expression of the *CDH13c*, *CDH13e*, *PTPRDa*, and *PTPRDd* transcripts was observed. These appear to be prematurely terminated transcripts and may be a general indication that cocaine is altering RNA transcription of these genes. As with the sensitization data, none of these changes were terribly large, but again, this is not surprising since these changes were likely to have occurred in a relatively small subset of neurons within each of these brain regions and perhaps only within certain portions of the dissected regions.

Although these data are quite preliminary, they do support a role for these CAMs in the underlying cellular changes that occur in response to repeated cocaine treatments, including contextual learning associated with drug seeking as measured in the CPP procedure.

### 2.6. Implications of These Findings for the Role of Cell Adhesion Molecules in Addiction

Prior to the emergence of so many associations between markers in CAM genes and drug dependence in GWAS studies, these genes were not at all considered to be important in addiction or in the mechanisms underlying responses to addictive drugs. This is not surprising as little was known about the role of these genes in most neural functions prior to these studies. A growing appreciation has developed for the role of cell adhesion molecules not only in neural development, but also in neuroplasticity occurring throughout the lifespan.

A very important issue regarding the role of CAMs in addiction involves the cellular and anatomical distribution of CAMs, and whether these are found in regions of the brain that are likely to influence addiction phenotypes. For many of the CAMs discussed here, there is certainly evidence for localization in excitatory and inhibitory synapses, but there is certainly much work to be done to identify which particular CAMs associate with which synapses, as well as the specific regional distribution of CAMs. A complete consideration of this topic is beyond the scope of this review, but it has been noted that many of the cell adhesion molecules discussed here are located in portions of corticostriatal circuitry involved in addiction on dopaminergic or glutamatergic neurons including CDH13, PTPRD, NLGN1, and NRXN3 [9]. It will be very important to separate the potential developmental roles of these genes from their roles in adult plasticity as well. For one addiction-associated CAM, CDH13 [98], a conditional knockout strategy, has suggested that the role of CDH13 influences adult plasticity. It is likely that many CAMs have developmental roles, or roles in both developmental and adult neural plasticity.

Not only have human studies repeatedly demonstrated the involvement of CAM variation in addiction, but mouse studies have now supported these findings. Studies in genetically modified mice have shown that reductions in the expression of several CAM genes, including NRCAM [44], CDH13 [98], CSMD1 [149], and PTPRD [125], affect responses to drugs of abuse, particularly cocaine, in standard animal models of psychostimulant responses that are important in the study of addictive properties of abused drugs. The levels of NRXN3 have also been shown to be upregulated in the globus pallidus during cocaine abstinence in mice [115]. Mechanistically, several CAMs have been shown to play integral roles in both postsynaptic and presynaptic differentiation and assembly in systems thought to be essential for the neuroplasticity of addiction. CAMs such as PSA-NCAM and SYNCAM, as well as several neurexins and neuroligins, differentially affect synaptic functions demonstrated by alterations in NMDA and AMPA receptor-mediated currents, as well as the expression of synaptic protein-mediated aspects of excitatory and inhibitory neurotransmission. These functions, when affected by drug exposure, may produce important neuroplastic changes fundamental to the development of addiction phenotypes. Thus, preclinical data supports GWAS findings suggesting a role of these genes in addiction, and by implication, that neural plasticity during development or after exposure to drugs of abuse is fundamental to the influence of variation in the function of these genes on addictive processes. Although certainly much remains to be done in this nascent field, the data also suggests that these molecules should be explored as potential targets of therapeutic interventions [10].

## Figures and Tables

**Table 1:** Abbreviations used for cell adhesion molecules discussed in this review.

Cell adhesion molecule	Abbreviation
Neural cell adhesion molecule	NCAM
Polysialated neural cell adhesion molecule	PSA-NCAM
Neuronal cell adhesion molecule	NRCAM
Immunoglobulin super family	IGSF
Synaptic cell adhesion molecule	SYNCAM
Intercellular adhesion molecule 5	ICAM5
Cadherin 13	CDH13
Neurexin 3	NRXN3
Neurexin 2*β*	NRXN2*β*
Neuroligin 3	NLGN3
Neuroligan 1	NLGN1
Neurexin 3*β*	NRXN3*β*
Protein tyrosine phosphatase receptor D	PTPRD
Protein tyrosine phosphatase receptor B	PTPRB
CUB and Sushi multiple domains 1	CSMD1
Protein tyrosine phosphatase receptor Z 1	PTPRZ1

**Table 2:** Gene expression changes after repeated cocaine treatment.

	ST	vMB	CX	HC
RBFOX1a	0.99 ± 0.06	0.88 ± 0.05	0.94 ± 0.01^∗^	1.05 ± 0.08
RBFOX1f	0.96 ± 0.04	0.95 ± 0.04	1.02 ± 0.02	1.01 ± 0.10
CDH13a	1.14 ± 0.05^∗^	1.04 ± 0.03	1.27 ± 0.06^∗∗^	1.26 ± 0.09
CDH13c	1.01 ± 0.13	1.36 ± 0.14	0.87 ± 0.10	0.94 ± 0.10
CDH13e	0.90 ± 0.05	1.23 ± 0.13	1.10 ± 0.08	1.07 ± 0.07
CSMD1g	1.24 ± 0.06^∗^	1.06 ± 0.04	0.98 ± 0.05	1.17 ± 0.08
CSMD1h	1.10 ± 0.07	1.04 ± 0.08	0.92 ± 0.08	0.88 ± 0.06
PTPRDa	0.95 ± 0.03	1.02 ± 0.03	0.82 ± 0.03^∗∗^	0.98 ± 0.08
PtPRDd	1.39 ± 0.07^∗∗^	1.25 ± 0.09^∗^	1.17 ± 0.07	0.97 ± 0.19

Data expressed as fold change as compared to saline controls (*N* = 9–12 per experimental condition). ^∗^ *p* < 0.05; ^∗∗^ *p* < 0.005. Note that the differences that are nominally significant at the *p* < 0.05 level might be false positives, while those that are significant at the *p* < 0.005 level are significant after a Bonferroni correction.

**Table 3:** Gene expression changes after cocaine CPP.

	ST	vMB	CX	HC
RBFOX1-a	0.85 ± 0.02^∗∗^	0.95 ± 0.05	1.04 ± 0.03	0.98 ± 0.02
RBFOX1-f	0.96 ± 0.02	1.03 ± 0.08	1.04 ± 0.02	1.00 ± 0.04
CDH13a	1.09 ± 0.06	1.12 ± 0.04^∗^	1.14 ± 0.04^∗^	1.05 ± 0.06
CDH13c	0.93 ± 0.09	1.15 ± 0.06	—	0.76 ± 0.05^∗^
CDH13e	0.88 ± 0.04	1.04 ± 0.07	—	0.76 ± 0.04^∗^
CSMD1g	1.12 ± 0.06	1.17 ± 0.06^∗^	1.21 ± 0.05^∗^	0.98 ± 0.04
CSMD1h	1.07 ± 0.09	1.13 ± 0.08	1.29 ± 0.06^∗∗^	0.81 ± 0.07
PTPRDa	0.89 ± 0.03	0.94 ± 0.03	—	0.92 ± 0.02^∗^
PtPRDd	0.51 ± 0.05^∗∗^	1.01 ± 0.13	1.26 ± 0.14	0.71 ± 0.04^∗^

Data expressed as fold change as compared to saline controls (*N* = 9–12 per experimental condition). ^∗^ *p* < 0.05; ^∗∗^ *p* < 0.005. Note that the differences that are nominally significant at the *p* < 0.05 level might be false positives, while those that are significant at the *p* < 0.005 level are significant after a Bonferroni correction.

## Supplementary Materials

Supplementary Materials Supplemental Table S1: primers for rtPCR.

## References

[1] American Psychiatric Association. and American Psychiatric Association (2013). Diagnostic and statistical manual of mental disorders. *DSM-5*.

[2] Koob G. F., Le Moal M. (2008). Neurobiological mechanisms for opponent motivational processes in addiction. *Philosophical Transactions of the Royal Society B: Biological Sciences*.

[3] Hall F. S., Drgonova J., Jain S., Uhl G. R. (2013). Implications of genome wide association studies for addiction: are our a *priori* assumptions all wrong?. *Pharmacology & Therapeutics*.

[4] Agrawal A., Lynskey M. T. (2008). Are there genetic influences on addiction: evidence from family, adoption and twin studies. *Addiction*.

[5] Hall F. S., Preedy V. (2016). Reverse translational implications of genome-wide association studies for addiction genetics. *The Neuropathology of Drug Additions and Drug Misuse*.

[6] Drgon T., Johnson C. A., Nino M., Drgonova J., Walther D. M., Uhl G. R. (2011). “Replicated” genome wide association for dependence on illegal substances: genomic regions identified by overlapping clusters of nominally positive SNPs. *American Journal of Medical Genetics. Part B, Neuropsychiatric Genetics*.

[7] Drgon T., Zhang P. W., Johnson C. (2010). Genome wide association for addiction: replicated results and comparisons of two analytic approaches. *PLoS One*.

[8] Uhl G. R., Drgon T., Johnson C. (2008). “Higher order” addiction molecular genetics: convergent data from genome-wide association in humans and mice. *Biochemical Pharmacology*.

[9] Zhong X., Drgonova J., Li C. Y., Uhl G. R. (2015). Human cell adhesion molecules: annotated functional subtypes and overrepresentation of addiction-associated genes. *Annals of the New York Academy of Sciences*.

[10] Uhl G. R., Drgonova J. (2014). Cell adhesion molecules: druggable targets for modulating the connectome and brain disorders?. *Neuropsychopharmacology*.

[11] Uhl G. R., Drgonova J., Hall F. S. (2014). Curious cases: altered dose-response relationships in addiction genetics. *Pharmacology & Therapeutics*.

[12] Sytnyk V., Leshchyns'ka I., Schachner M. (2017). Neural cell adhesion molecules of the immunoglobulin superfamily regulate synapse formation, maintenance, and function. *Trends in Neurosciences*.

[13] Walsh F. S., Doherty P. (1997). Neural cell adhesion molecules of the immunoglobulin superfamily: role in axon growth and guidance. *Annual Review of Cell and Developmental Biology*.

[14] Harpaz Y., Chothia C. (1994). Many of the immunoglobulin superfamily domains in cell adhesion molecules and surface receptors belong to a new structural set which is close to that containing variable domains. *Journal of Molecular Biology*.

[15] Berezin V. (2009). Structure and function of the neural cell adhesion molecule NCAM. *Advances in Experimental Medicine and Biology*.

[16] Hortsch M. (2000). Structural and functional evolution of the L1 family: are four adhesion molecules better than one?. *Molecular and Cellular Neurosciences*.

[17] Biederer T. (2005). Progress from the postsynaptic side: signaling in synaptic differentiation. *Science Signaling*.

[18] Walsh F. S., Doherty P. (1991). Structure and function of the gene for neural cell adhesion molecule. *Seminars in Neuroscience*.

[19] Hoffman S., Sorkin B. C., White P. C. (1982). Chemical characterization of a neural cell adhesion molecule purified from embryonic brain membranes. *The Journal of Biological Chemistry*.

[20] Cremer H., Chazal G., Carleton A., Goridis C., Vincent J. D., Lledo P. M. (1998). Long-term but not short-term plasticity at mossy fiber synapses is impaired in neural cell adhesion molecule-deficient mice. *Proceedings of the National Academy of Sciences of the United States of America*.

[21] Luthi A., Laurent J. P., Figurov A., Muller D., Schachner M. (1994). Hippocampal long-term potentiation and neural cell adhesion molecules L1 and Ncam. *Nature*.

[22] Muller D., Djebbara-Hannas Z., Jourdain P. (2000). Brain-derived neurotrophic factor restores long-term potentiation in polysialic acid-neural cell adhesion molecule-deficient hippocampus. *Proceedings of the National Academy of Sciences of the United States of America*.

[23] Stoenica L., Senkov O., Gerardy-Schahn R., Weinhold B., Schachner M., Dityatev A. (2006). *In vivo* synaptic plasticity in the dentate gyrus of mice deficient in the neural cell adhesion molecule NCAM or its polysialic acid. *The European Journal of Neuroscience*.

[24] Bukalo O., Fentrop N., Lee A. Y. (2004). Conditional ablation of the neural cell adhesion molecule reduces precision of spatial learning, long-term potentiation, and depression in the CA1 subfield of mouse hippocampus. *The Journal of Neuroscience*.

[25] Muller D., Wang C., Skibo G. (1996). PSA-NCAM is required for activity-induced synaptic plasticity. *Neuron*.

[26] Guiraudie-Capraz G., Chaillan F. A., Truchet B., Franc J. L., Mourre C., Roman F. S. (2011). Increase in polysialyltransferase gene expression following LTP in adult rat dentate gyrus. *Hippocampus*.

[27] Sytnyk V., Leshchyns'ka I., Nikonenko A. G., Schachner M. (2006). NCAM promotes assembly and activity-dependent remodeling of the postsynaptic signaling complex. *The Journal of Cell Biology*.

[28] Vaithianathan T., Matthias K., Bahr B. (2004). Neural cell adhesion molecule-associated polysialic acid potentiates *α*-amino-3-hydroxy-5-methylisoxazole-4-propionic acid receptor currents. *The Journal of Biological Chemistry*.

[29] Hammond M. S., Sims C., Parameshwaran K., Suppiramaniam V., Schachner M., Dityatev A. (2006). Neural cell adhesion molecule-associated polysialic acid inhibits NR2B-containing N-methyl-D-aspartate receptors and prevents glutamate-induced cell death. *The Journal of Biological Chemistry*.

[30] Mackowiak M., Grzegorzewska M., Budziszewska B., Chocyk A., Hess G., Wedzony K. (2008). Cocaine decreases the expression of PSA-NCAM protein and attenuates long-term potentiation via glucocorticoid receptors in the rat dentate gyrus. *The European Journal of Neuroscience*.

[31] Heidmets L. T., Kalda A., Zharkovsky A. (2007). Acute amphetamine treatment decreases the expression of 180-200 kDa isoform of polysialic acid linked neural cell adhesion molecule in mouse hippocampus. *Brain Research*.

[32] Mackowiak M., Chocyk A., Dudys D., Wedzony K. (2009). Activation of CB1 cannabinoid receptors impairs memory consolidation and hippocampal polysialylated neural cell adhesion molecule expression in contextual fear conditioning. *Neuroscience*.

[33] Abrous D. N., Adriani W., Montaron M. F. (2002). Nicotine self-administration impairs hippocampal plasticity. *The Journal of Neuroscience*.

[34] Barker J. M., Torregrossa M. M., Taylor J. R. (2012). Low prefrontal PSA-NCAM confers risk for alcoholism-related behavior. *Nature Neuroscience*.

[35] Weber M., Modemann S., Schipper P. (2006). Increased polysialic acid neural cell adhesion molecule expression in human hippocampus of heroin addicts. *Neuroscience*.

[36] Cao J., Dwyer J. B., Mangold J. E. (2011). Modulation of cell adhesion systems by prenatal nicotine exposure in limbic brain regions of adolescent female rats. *The International Journal of Neuropsychopharmacology*.

[37] Stringer S., Minică C. C., Verweij K. J. H. (2016). Genome-wide association study of lifetime cannabis use based on a large meta-analytic sample of 32 330 subjects from the International Cannabis Consortium. *Translational Psychiatry*.

[38] Gelernter J., Yu Y., Weiss R. (2006). Haplotype spanning *TTC12* and *ANKK1*, flanked by the *DRD2* and *NCAM1* loci, is strongly associated to nicotine dependence in two distinct American populations. *Human Molecular Genetics*.

[39] Bidwell L. C., McGeary J. E., Gray J. C., Palmer R. H. C., Knopik V. S., MacKillop J. (2015). *NCAM1-TTC12-ANKK1-DRD2* variants and smoking motives as intermediate phenotypes for nicotine dependence. *Psychopharmacology*.

[40] Ducci F., Kaakinen M., Pouta A. (2011). *TTC12-ANKK1-DRD2* and *CHRNA5-CHRNA3-CHRNB4* influence different pathways leading to smoking behavior from adolescence to mid-adulthood. *Biological Psychiatry*.

[41] Yang B. Z., Kranzler H. R., Zhao H., Gruen J. R., Luo X., Gelernter J. (2007). Association of haplotypic variants in *DRD2*, *ANKK1*, *TTC12* and *NCAM1* to alcohol dependence in independent casecontrol and family samples. *Human Molecular Genetics*.

[42] Grumet M. (1997). Nr-CAM: a cell adhesion molecule with ligand and receptor functions. *Cell and Tissue Research*.

[43] Sakurai T. (2012). The role of NrCAM in neural development and disorders–beyond a simple glue in the brain. *Molecular and Cellular Neurosciences*.

[44] Ishiguro H., Liu Q. R., Gong J. P. (2006). *NrCAM* in addiction vulnerability: positional cloning, drug-regulation, haplotype-specific expression, and altered drug reward in knockout mice. *Neuropsychopharmacology*.

[45] Reich T. (1996). A genomic survey of alcohol dependence and related phenotypes: results from the Collaborative Study on the Genetics of Alcoholism (COGA). *Alcoholism, Clinical and Experimental Research*.

[46] Reich T., Edenberg H. J., Goate A. (1998). Genome-wide search for genes affecting the risk for alcohol dependence. *American Journal of Medical Genetics*.

[47] Long J. C., Knowler W. C., Hanson R. L. (1998). Evidence for genetic linkage to alcohol dependence on chromosomes 4 and 11 from an autosome-wide scan in an American Indian population. *American Journal of Medical Genetics*.

[48] Uhl G. R., Liu Q. R., Walther D., Hess J., Naiman D. (2001). Polysubstance abuse-vulnerability genes: genome scans for association, using 1,004 subjects and 1,494 single-nucleotide polymorphisms. *American Journal of Human Genetics*.

[49] Uhl G. R., Liu Q. R., Naiman D. (2002). Substance abuse vulnerability loci: converging genome scanning data. *Trends in Genetics*.

[50] Yoo B. K., Shim J. C., Lee B. D. (2012). Association of the neuronal cell adhesion molecule (NrCAM) gene variants with personality traits and addictive symptoms in methamphetamine use disorder. *Psychiatry Investigation*.

[51] Ishiguro H., Hall F. S., Horiuchi Y. (2014). NrCAM-regulating neural systems and addiction-related behaviors. *Addiction Biology*.

[52] Matzel L. D., Babiarz J., Townsend D. A., Grossman H. C., Grumet M. (2008). Neuronal cell adhesion molecule deletion induces a cognitive and behavioral phenotype reflective of impulsivity. *Genes, Brain, and Behavior*.

[53] Frei J. A., Stoeckli E. T. (2017). SynCAMs - from axon guidance to neurodevelopmental disorders. *Molecular and Cellular Neurosciences*.

[54] Frei J. A., Stoeckli E. T. (2014). SynCAMs extend their functions beyond the synapse. *The European Journal of Neuroscience*.

[55] Biederer T., Sara Y., Mozhayeva M. (2002). SynCAM, a synaptic adhesion molecule that drives synapse assembly. *Science*.

[56] Scheiffele P., Fan J., Choih J., Fetter R., Serafini T. (2000). Neuroligin expressed in nonneuronal cells triggers presynaptic development in contacting axons. *Cell*.

[57] Galuska S. P., Rollenhagen M., Kaup M. (2010). Synaptic cell adhesion molecule SynCAM 1 is a target for polysialylation in postnatal mouse brain. *Proceedings of the National Academy of Sciences of the United States of America*.

[58] Robinson T. E., Gorny G., Mitton E., Kolb B. (2001). Cocaine self-administration alters the morphology of dendrites and dendritic spines in the nucleus accumbens and neocortex. *Synapse*.

[59] Robinson T. E., Gorny G., Savage V. R., Kolb B. (2002). Widespread but regionally specific effects of experimenter- versus self-administered morphine on dendritic spines in the nucleus accumbens, hippocampus, and neocortex of adult rats. *Synapse*.

[60] Robinson T. E., Kolb B. (1999). Alterations in the morphology of dendrites and dendritic spines in the nucleus accumbens and prefrontal cortex following repeated treatment with amphetamine or cocaine. *The European Journal of Neuroscience*.

[61] Boudreau A. C., Wolf M. E. (2005). Behavioral sensitization to cocaine is associated with increased AMPA receptor surface expression in the nucleus accumbens. *The Journal of Neuroscience*.

[62] Boudreau A. C., Reimers J. M., Milovanovic M., Wolf M. E. (2007). Cell surface AMPA receptors in the rat nucleus accumbens increase during cocaine withdrawal but internalize after cocaine challenge in association with altered activation of mitogen-activated protein kinases. *The Journal of Neuroscience*.

[63] Mead A. N., Zamanillo D., Becker N., Stephens D. N. (2007). AMPA-receptor GluR1 subunits are involved in the control over behavior by cocaine-paired cues. *Neuropsychopharmacology*.

[64] Giza J. I., Jung Y., Jeffrey R. A., Neugebauer N. M., Picciotto M. R., Biederer T. (2013). The synaptic adhesion molecule SynCAM 1 contributes to cocaine effects on synapse structure and psychostimulant behavior. *Neuropsychopharmacology*.

[65] Gahmberg C. G., Ning L., Paetau S. (2014). ICAM-5: a neuronal dendritic adhesion molecule involved in immune and neuronal functions. *Advances in Neurobiology*.

[66] Gahmberg C. G., Tian L., Ning L., Nyman-Huttunen H. (2008). ICAM-5—a novel two-facetted adhesion molecule in the mammalian brain. *Immunology Letters*.

[67] Lonskaya I., Partridge J., Lalchandani R. R. (2013). Soluble ICAM-5, a product of activity dependent proteolysis, increases mEPSC frequency and dendritic expression of GluA1. *PLoS One*.

[68] Tian L., Stefanidakis M., Ning L. (2007). Activation of NMDA receptors promotes dendritic spine development through MMP-mediated ICAM-5 cleavage. *The Journal of Cell Biology*.

[69] Conant K., Lonskaya I., Szklarczyk A. (2011). Methamphetamine-associated cleavage of the synaptic adhesion molecule intercellular adhesion molecule-5. *Journal of Neurochemistry*.

[70] Stefaniuk M., Beroun A., Lebitko T. (2017). Matrix Metalloproteinase-9 and synaptic plasticity in the central amygdala in control of alcohol-seeking behavior. *Biological Psychiatry*.

[71] Fernandes S., Salta S., Bravo J., Silva A. P., Summavielle T. (2016). Acetyl-L-carnitine prevents methamphetamine-induced structural damage on endothelial cells via ILK-related MMP-9 activity. *Molecular Neurobiology*.

[72] Wright J. W., Harding J. W. (2009). Contributions of matrix metalloproteinases to neural plasticity, habituation, associative learning and drug addiction. *Neural Plasticity*.

[73] Yamada K. (2008). Endogenous modulators for drug dependence. *Biological and Pharmaceutical Bulletin*.

[74] Samochowiec A., Grzywacz A., Kaczmarek L. (2010). Functional polymorphism of matrix metalloproteinase-9 (MMP-9) gene in alcohol dependence: family and case control study. *Brain Research*.

[75] Mash D. C., ffrench-Mullen J., Adi N., Qin Y., Buck A., Pablo J. (2007). Gene expression in human hippocampus from cocaine abusers identifies genes which regulate extracellular matrix remodeling. *PLoS One*.

[76] Hirano S., Takeichi M. (2012). Cadherins in brain morphogenesis and wiring. *Physiological Reviews*.

[77] Takeichi M. (1988). The cadherins: cell-cell adhesion molecules controlling animal morphogenesis. *Development*.

[78] Yoshida C., Takeichi M. (1982). Teratocarcinoma cell adhesion: identification of a cell-surface protein involved in calcium-dependent cell aggregation. *Cell*.

[79] Nagafuchi A., Takeichi M. (1988). Cell binding function of E-cadherin is regulated by the cytoplasmic domain. *The EMBO Journal*.

[80] Hoschuetzky H., Aberle H., Kemler R. (1994). Beta-catenin mediates the interaction of the cadherin-catenin complex with epidermal growth factor receptor. *The Journal of Cell Biology*.

[81] Hulsken J., Birchmeier W., Behrens J. (1994). E-cadherin and APC compete for the interaction with beta-catenin and the cytoskeleton. *The Journal of Cell Biology*.

[82] Oyama T., Kanai Y., Ochiai A. (1994). A truncated *β*-catenin disrupts the interaction between E-cadherin and *α*-catenin: a cause of loss of intercellular adhesiveness in human cancer cell lines. *Cancer Research*.

[83] Hirano K., Kaneko R., Izawa T., Kawaguchi M., Kitsukawa T., Yagi T. (2012). Single-neuron diversity generated by *Protocadherin-β* cluster in mouse central and peripheral nervous systems. *Frontiers in Molecular Neuroscience*.

[84] Treutlein J., Cichon S., Ridinger M. (2009). Genome-wide association study of alcohol dependence. *Archives of General Psychiatry*.

[85] Uhl G. R., Drgon T., Johnson C. (2008). Molecular genetics of addiction and related heritable phenotypes: genome-wide association approaches identify “connectivity constellation” and drug target genes with pleiotropic effects. *Annals of the New York Academy of Sciences*.

[86] Thorgeirsson T. E., Gudbjartsson D. F., Surakka I. (2010). Sequence variants at *CHRNB3-CHRNA6* and *CYP2A6* affect smoking behavior. *Nature Genetics*.

[87] Uhl G. R., Drgon T., Liu Q. R. (2008). Genome-wide association for methamphetamine dependence: convergent results from 2 samples. *Archives of General Psychiatry*.

[88] Johnson C., Drgon T., Walther D., Uhl G. R. (2011). Genomic regions identified by overlapping clusters of nominally-positive SNPs from genome-wide studies of alcohol and illegal substance dependence. *PLoS One*.

[89] Liu Q. R., Drgon T., Johnson C., Walther D., Hess J., Uhl G. R. (2006). Addiction molecular genetics: 639,401 SNP whole genome association identifies many "cell adhesion" genes. *American Journal of Medical Genetics. Part B, Neuropsychiatric Genetics*.

[90] Johnson C., Drgon T., Liu Q. R. (2006). Pooled association genome scanning for alcohol dependence using 104,268 SNPs: validation and use to identify alcoholism vulnerability loci in unrelated individuals from the collaborative study on the genetics of alcoholism. *American Journal of Medical Genetics Part B, Neuropsychiatric Genetics*.

[91] Johnson C., Drgon T., Liu Q. R. (2008). Genome wide association for substance dependence: convergent results from epidemiologic and research volunteer samples. *BMC Medical Genetics*.

[92] Drgon T., Montoya I., Johnson C. (2009). Genome-wide association for nicotine dependence and smoking cessation success in NIH research volunteers. *Molecular Medicine*.

[93] Hall F. S., Markou A., Levin E. D., Uhl G. R. (2012). Mouse models for studying genetic influences on factors determining smoking cessation success in humans. *Annals of the New York Academy of Sciences*.

[94] Hart A. B., Engelhardt B. E., Wardle M. C. (2012). Genome-wide association study of *d*-amphetamine response in healthy volunteers identifies putative associations, including cadherin 13 (*CDH13*). *PLoS One*.

[95] Joslyn G., Ravindranathan A., Brush G., Schuckit M., White R. L. (2010). Human variation in alcohol response is influenced by variation in neuronal signaling genes. *Alcoholism, Clinical & Experimental Research*.

[96] Ranscht B., Dours-Zimmermann M. T. (1991). T-cadherin, a novel cadherin cell adhesion molecule in the nervous system lacks the conserved cytoplasmic region. *Neuron*.

[97] Vestal D. J., Ranscht B. (1992). Glycosyl phosphatidylinositol--anchored T-cadherin mediates calcium-dependent, homophilic cell adhesion. *The Journal of Cell Biology*.

[98] Drgonova J., Walther D. (2016). Cadherin 13: human cis-regulation and selectively-altered addiction phenotypes and cerebral cortical dopamine in knockout mice. *Molecular Medicine*.

[99] Bierut L. J., Madden P. A. F., Breslau N. (2007). Novel genes identified in a high-density genome wide association study for nicotine dependence. *Human Molecular Genetics*.

[100] Rudenko G. (2017). Dynamic control of synaptic adhesion and organizing molecules in synaptic plasticity. *Neural Plasticity*.

[101] Bang M. L., Owczarek S. (2013). A matter of balance: role of neurexin and neuroligin at the synapse. *Neurochemical Research*.

[102] Lise M. F., El-Husseini A. (2006). The neuroligin and neurexin families: from structure to function at the synapse. *Cellular and Molecular Life Sciences*.

[103] Craig A. M., Kang Y. (2007). Neurexin-neuroligin signaling in synapse development. *Current Opinion in Neurobiology*.

[104] Dean C., Dresbach T. (2006). Neuroligins and neurexins: linking cell adhesion, synapse formation and cognitive function. *Trends in Neurosciences*.

[105] Liu Q. R., Drgon T., Walther D. (2005). Pooled association genome scanning: validation and use to identify addiction vulnerability loci in two samples. *Proceedings of the National Academy of Sciences of the United States of America*.

[106] Lachman H. M., Fann C. S. J., Bartzis M. (2007). Genomewide suggestive linkage of opioid dependence to chromosome 14q. *Human Molecular Genetics*.

[107] Docampo E., Ribasés M., Gratacòs M. (2012). Association of neurexin 3 polymorphisms with smoking behavior. *Genes, Brain, and Behavior*.

[108] Hishimoto A., Liu Q. R., Drgon T. (2007). Neurexin 3 polymorphisms are associated with alcohol dependence and altered expression of specific isoforms. *Human Molecular Genetics*.

[109] Stoltenberg S. F., Lehmann M. K., Christ C. C., Hersrud S. L., Davies G. E. (2011). Associations among types of impulsivity, substance use problems and *Neurexin*-3 polymorphisms. *Drug and Alcohol Dependence*.

[110] Novak G., Boukhadra J., Shaikh S. A., Kennedy J. L., Le Foll B. (2009). Association of a polymorphism in the NRXN3 gene with the degree of smoking in schizophrenia: a preliminary study. *The World Journal of Biological Psychiatry*.

[111] Heine M., Thoumine O., Mondin M., Tessier B., Giannone G., Choquet D. (2008). Activity-independent and subunit-specific recruitment of functional AMPA receptors at neurexin/neuroligin contacts. *Proceedings of the National Academy of Sciences of the United States of America*.

[112] Kim S., Burette A., Chung H. S. (2006). NGL family PSD-95–interacting adhesion molecules regulate excitatory synapse formation. *Nature Neuroscience*.

[113] Poulopoulos A., Aramuni G., Meyer G. (2009). Neuroligin 2 drives postsynaptic assembly at perisomatic inhibitory synapses through gephyrin and collybistin. *Neuron*.

[114] Zhang C., Atasoy D., Araç D. (2010). Neurexins physically and functionally interact with GABA_A_ receptors. *Neuron*.

[115] Kelai S., Maussion G., Noble F. (2008). Nrxn3 upregulation in the globus pallidus of mice developing cocaine addiction. *NeuroReport*.

[116] Selner N. G., Luechapanichkul R., Chen X. (2014). Diverse levels of sequence selectivity and catalytic efficiency of protein-tyrosine phosphatases. *Biochemistry*.

[117] Drgon T., Johnson C., Walther D., Albino A. P., Rose J. E., Uhl G. R. (2009). Genome-wide association for smoking cessation success: participants in a trial with adjunctive denicotinized cigarettes. *Molecular Medicine*.

[118] Uhl G. R., Drgon T., Johnson C., Ramoni M. F., Behm F. M., Rose J. E. (2010). Genome-wide association for smoking cessation success in a trial of precessation nicotine replacement. *Molecular Medicine*.

[119] Uhl G. R., Drgon T., Johnson C. (2010). Genome-wide association for smoking cessation success: participants in the patch in practice trial of nicotine replacement. *Pharmacogenomics*.

[120] Uhl G. R., Walther D., Musci R. (2014). Smoking quit success genotype score predicts quit success and distinct patterns of developmental involvement with common addictive substances. *Molecular Psychiatry*.

[121] Li D., Zhao H., Kranzler H. R. (2015). Genome-wide association study of copy number variations (CNVs) with opioid dependence. *Neuropsychopharmacology*.

[122] Lein E. S., Hawrylycz M. J., Ao N. (2007). Genome-wide atlas of gene expression in the adult mouse brain. *Nature*.

[123] Wang J., Bixby J. L. (1999). Receptor tyrosine phosphatase-δ is a homophilic, neurite-promoting cell adhesion molecule for CNS neurons. *Molecular and Cellular Neurosciences*.

[124] Yim Y. S., Kwon Y., Nam J. (2013). Slitrks control excitatory and inhibitory synapse formation with LAR receptor protein tyrosine phosphatases. *Proceedings of the National Academy of Sciences of the United States of America*.

[125] Drgonova J., Walther D. (2015). Mouse model for protein tyrosine phosphatase D (*PTPRD*) associations with restless leg syndrome or Willis-Ekbom disease and addiction: reduced expression alters locomotion, sleep behaviors and cocaine-conditioned place preference. *Molecular Medicine*.

[126] Stoker A. W. (2015). RPTPs in axons, synapses and neurology. *Seminars in Cell & Developmental Biology*.

[127] Ishiguro H., Gong J. P., Hall F. S., Arinami T., Uhl G. R. (2008). Association of *PTPRB* gene polymorphism with drug addiction. *American Journal of Medical Genetics Part B: Neuropsychiatric Genetics*.

[128] Garcia-Perez D., Laorden M. L., Milanes M. V. (2017). Acute morphine, chronic morphine, and morphine withdrawal differently affect pleiotrophin, midkine, and receptor protein tyrosine phosphatase *β*/*ζ* regulation in the ventral tegmental area. *Molecular Neurobiology*.

[129] Muramatsu T. (2002). Midkine and pleiotrophin: two related proteins involved in development, survival, inflammation and tumorigenesis. *The Journal of Biochemistry*.

[130] Mailleux P., Preud'homme X., Albala N., Vanderwinden J. M., Vanderhaeghen J. J. (1994). Δ-9-tetrahydrocannabinol regulates gene expression of the growth factor pleiotrophin in the forebrain. *Neuroscience Letters*.

[131] Le Greves P. (2005). Pleiotrophin gene transcription in the rat nucleus accumbens is stimulated by an acute dose of amphetamine. *Brain Research Bulletin*.

[132] Gramage E., Pérez-García C., Vicente-Rodríguez M., Bollen S., Rojo L., Herradón G. (2013). Regulation of extinction of cocaine-induced place preference by midkine is related to a differential phosphorylation of peroxiredoxin 6 in dorsal striatum. *Behavioural Brain Research*.

[133] Gramage E., Vicente-Rodriguez M., Herradon G. (2015). Pleiotrophin modulates morphine withdrawal but has no effects on morphine-conditioned place preference. *Neuroscience Letters*.

[134] Gramage E., Del Olmo N., Fole A., Martín Y. B., Herradón G. (2013). Periadolescent amphetamine treatment causes transient cognitive disruptions and long-term changes in hippocampal LTP depending on the endogenous expression of pleiotrophin. *Addiction Biology*.

[135] Vicente-Rodriguez M., Gramage E., Herradón G., Pérez-García C. (2013). Phosphoproteomic analysis of the striatum from pleiotrophin knockout and midkine knockout mice treated with cocaine reveals regulation of oxidative stress-related proteins potentially underlying cocaine-induced neurotoxicity and neurodegeneration. *Toxicology*.

[136] Gramage E., Alguacil L. F., Herradon G. (2008). Pleiotrophin prevents cocaine-induced toxicity in vitro. *European Journal of Pharmacology*.

[137] Soto-Montenegro M. L., Vicente-Rodríguez M., Pérez-García C., Gramage E., Desco M., Herradón G. (2015). Functional neuroimaging of amphetamine-induced striatal neurotoxicity in the pleiotrophin knockout mouse model. *Neuroscience Letters*.

[138] Chen G., Zhang F., Xue W. (2016). An association study revealed substantial effects of dominance, epistasis and substance dependence co-morbidity on alcohol dependence symptom count. *Addiction Biology*.

[139] Kraus D. M., Elliott G. S., Chute H. (2006). CSMD1 is a novel multiple domain complement-regulatory protein highly expressed in the central nervous system and epithelial tissues. *The Journal of Immunology*.

[140] Sherva R., Wang Q., Kranzler H. (2016). Genome-wide association study of cannabis dependence severity, novel risk variants, and shared genetic risks. *JAMA Psychiatry*.

[141] Yin X. Y., Cheng H., Wang W. J. (2013). *TNIP1/ANXA6* and *CSMD1* variants interacting with cigarette smoking, alcohol intake affect risk of psoriasis. *Journal of Dermatological Science*.

[142] Urashima M., Hama T., Suda T. (2013). Distinct effects of alcohol consumption and smoking on genetic alterations in head and neck carcinoma. *PLoS One*.

[143] Havik B., Le Hellard S., Rietschel M. (2011). The complement control-related genes *CSMD1* and *CSMD2* associate to schizophrenia. *Biological Psychiatry*.

[144] Cukier H. N., Dueker N. D., Slifer S. H. (2014). Exome sequencing of extended families with autism reveals genes shared across neurodevelopmental and neuropsychiatric disorders. *Molecular Autism*.

[145] Xu W., Cohen-Woods S., Chen Q. (2014). Genome-wide association study of bipolar disorder in Canadian and UK populations corroborates disease loci including *SYNE1* and *CSMD1*. *BMC Medical Genetics*.

[146] Koiliari E., Roussos P., Pasparakis E. (2014). The CSMD1 genome-wide associated schizophrenia risk variant rs10503253 affects general cognitive ability and executive function in healthy males. *Schizophrenia Research*.

[147] Distler M. G., Opal M. D., Dulawa S. C., Palmer A. A. (2012). Assessment of behaviors modeling aspects of schizophrenia in *Csmd1* mutant mice. *PLoS One*.

[148] Steen V. M., Nepal C., Ersland K. M. (2013). Neuropsychological deficits in mice depleted of the schizophrenia susceptibility gene *CSMD1*. *PLoS One*.

[149] Drgonova J., Walther D., Singhal S. (2015). Altered *CSMD1* expression alters cocaine-conditioned place preference: mutual support for a complex locus from human and mouse models. *PLoS One*.

[150] Kiehl T. R., Shibata H., Vo T., Huynh D. P., Pulst S. M. (2001). Identification and expression of a mouse ortholog of A2BP1. *Mammalian Genome*.

[151] Shibata H., Huynh D. P., Pulst S. M. (2000). A novel protein with RNA-binding motifs interacts with ataxin-2. *Human Molecular Genetics*.

[152] Lee J. A., Tang Z. Z., Black D. L. (2009). An inducible change in Fox-1/A2BP1 splicing modulates the alternative splicing of downstream neuronal target exons. *Genes & Development*.

[153] Zhang C., Zhang Z., Castle J. (2008). Defining the regulatory network of the tissue-specific splicing factors Fox-1 and Fox-2. *Genes & Development*.

[154] Nakahata S., Kawamoto S. (2005). Tissue-dependent isoforms of mammalian Fox-1 homologs are associated with tissue-specific splicing activities. *Nucleic Acids Research*.

[155] Gehman L. T., Stoilov P., Maguire J. (2011). The splicing regulator Rbfox1 (A2BP1) controls neuronal excitation in the mammalian brain. *Nature Genetics*.

[156] Fogel B. L., Wexler E., Wahnich A. (2012). *RBFOX1* regulates both splicing and transcriptional networks in human neuronal development. *Human Molecular Genetics*.

[157] Hammock E. A., Levitt P. (2011). Developmental expression mapping of a gene implicated in multiple neurodevelopmental disorders, *A2bp1 (Fox1)*. *Developmental Neuroscience*.

[158] Voineagu I., Wang X., Johnston P. (2011). Transcriptomic analysis of autistic brain reveals convergent molecular pathology. *Nature*.

[159] Barnby G., Abbott A., Sykes N. (2005). Candidate-gene screening and association analysis at the autism-susceptibility locus on chromosome 16p: evidence of association at *GRIN2A* and *ABAT*. *American Journal of Human Genetics*.

[160] Uhl G. R., Liu Q. R., Drgon T. (2008). Molecular genetics of successful smoking cessation: convergent genome-wide association study results. *Archives of General Psychiatry*.

[161] Uhl G. R., Liu Q. R., Drgon T., Johnson C., Walther D., Rose J. E. (2007). Molecular genetics of nicotine dependence and abstinence: whole genome association using 520,000 SNPs. *BMC Genetics*.

[162] Ehlers C. L., Gilder D. A., Wall T. L., Phillips E., Feiler H., Wilhelmsen K. C. (2004). Genomic screen for loci associated with alcohol dependence in mission Indians. *American Journal of Medical Genetics Part B, Neuropsychiatric Genetics*.

[163] Morley K. I., Medland S. E., Ferreira M. A. R. (2006). A possible smoking susceptibility locus on chromosome 11p12: evidence from sex-limitation linkage analyses in a sample of Australian twin families. *Behavior Genetics*.

[164] Feng J., Wilkinson M., Liu X. (2014). Chronic cocaine-regulated epigenomic changes in mouse nucleus accumbens. *Genome Biology*.

