# Supplementary material for: The Role of Cell Adhesion Molecule Genes Regulating Neuroplasticity in Addiction
Source: Neural Plast. 2018 Feb 20;2018:9803764. doi: 10.1155/2018/9803764 (PMC5838467; doi:10.1155/2018/9803764)
Supplement: Supplementary Materials — Supplemental Table S1: primers for rtPCR. [file 9803764.f1.pdf]

Supplementary Information

Cocaine effects on gene expression in WT mice

Experimental Design: The effects of cocaine on gene expression were examined in tissue samples collected after a sensitizing regimen of cocaine and after cocaine conditioning in a standard conditioned place preference procedure. Based on the combined human and mouse data, 4 genes were nominated for analysis: *Rbfox1*, *Cdh13*, *Csmd1*, and *Ptprd*. The expression levels were calculated from relative calibration curves and all the genes were normalized to the geometrical average of three housekeeping genes (*Gapdh*, *Hprt*, and *Ubc*). The primer sequences are listed in Table S1, below.

Repeated cocaine treatments: *Sensitizing Cocaine regimen*. Male C57Bl/6j mice were chronically treated with cocaine (10 mg/kg, s.c.) or saline (10 mL/kg) for 5 days. Four days after the last injection a test dose was administered. After each injection mice were placed into 42 x 42 cm Plexiglas boxes for 20 min. Mice were euthanized 24 hr after the final cocaine injection by cervical dislocation and decapitation, brains were rapidly removed, dissected on an ice-cold plate, and mRNA isolated using the RNeasy lipid tissue mini kit (Qiagen). cDNA was synthetized with SuperScript III first strand synthesis supermix (Invitrogen) and levels of mRNA were assessed by quantitative RT-PCR with SybrGreen master mix (Applied Biosystems) according to the manufacturer's protocol. *Conditioned place preference*. Cocaine reward was assessed with the conditioned place preference (CPP) procedure using a two-compartment Plexiglas chamber. After two 20 min pre-tests in which subjects had access to both sides of the apparatus, two 20 min conditioning sessions over each of 2 days confined subjects injected with cocaine to the

compartment with a wire mesh floor and subjects injected with saline to the side with corncob bedding. Control subjects received saline injections on both sides. 24 hours after the last conditioning session, subjects were again given access to both sides of the apparatus for a 20 min post-test. The preference score was calculated as the difference between time spent on the drug-paired wire mesh during the post-test and the average time spent on the wire side during the pret-tests. Mice were euthanized 24 hr after the conditioning test by cervical dislocation and decapitation, brains were rapidly removed, dissected on an ice-cold plate, and mRNA isolated using the RNeasy lipid tissue mini kit (Qiagen). cDNA was synthetized with SuperScript III first strand synthesis supermix (Invitrogen) and levels of mRNA were assessed by quantitative RT-PCR with SybrGreen master mix (Applied Biosystems) according to the manufacturer's protocol.

Supplemental Table S1: Primers for rtPCR

	Forward Primer	Reverse Primer
RBFOX1a	TGGTTATGCTGCGTACCGCTA	GGCAGCATAAACTCGTCCGTA
RBFOX1f	TGGTTATGCTGCGTACCGCTA	TGTTGCAACGAAGACGAACTGA
CDH13a	CTTCCCCAGAGATGTCGGCA	AGCGATCGTTTCTCTGTCCAG
CDH13c	AGAGAAACGATCGCTACTTATCA	TGGCAGACCGCATGTGACCT
CDH13e	CTTCCCCAGAGATGTCGGCA	GAGCAGGCACCCAGGACTC
CSMD1g	GGCTCCTCACTGCAGCTAAG	CCATGAGGAAAGCCAGGACTT
CSMD1h	GGCTCCTCACTGCAGCTAAG	TTCAGTGGCACCCAAATATGTC
PTPRDa	ATGTCAGAGAGCTGCGAGAAG	TCCTGGAGGCTTGGGTAAGG
PtPRDd	GTGACCTTGTGTTTTGTTCCAG	TCCTGGAGGCTTGGGTAAGG
GAPDH	GCATGGCCTTCCGTGTTC	CACCACCTTCTTGATGTCATC
HPRT	GACACTGGTAAAACAATGCAAAC	GAGGTCCTTTTCACCAGCAAG
UBC	GCAGGCAAGCAGCTGGAAGA	TTCACAAAGATCTGCATCCCAC
